# Overexpression of a Neuronal Type Adenylyl Cyclase (Type 8) in Sinoatrial Node Markedly Impacts Heart Rate and Rhythm

**DOI:** 10.3389/fnins.2019.00615

**Published:** 2019-06-18

**Authors:** Jack M. Moen, Michael G. Matt, Christopher Ramirez, Kirill V. Tarasov, Khalid Chakir, Yelena S. Tarasova, Yevgeniya Lukyanenko, Kenta Tsutsui, Oliver Monfredi, Christopher H. Morrell, Syevda Tagirova, Yael Yaniv, Thanh Huynh, Karel Pacak, Ismayil Ahmet, Edward G. Lakatta

**Affiliations:** ^1^Intramural Research Program, Laboratory of Cardiovascular Science, National Institute on Aging, National Institutes of Health, Baltimore, MD, United States; ^2^Cellular and Molecular Physiology, Yale University, New Haven, CT, United States; ^3^School of Medicine, University of Pittsburgh, Pittsburgh, PA, United States; ^4^Department of Cardiovascular and Electrophysiology, The Johns Hopkins Hospital, Baltimore, MD, United States; ^5^Department of Mathematics, Loyola University Maryland, Baltimore, MD, United States; ^6^Faculty of Biomedical Engineering, Technion Israel Institute of Technology, Haifa, Israel; ^7^Section on Medical Neuroendocrinology, Eunice Kennedy Shriver National Institute of Child Health and Human Development, National Institutes of Health, Bethesda, MD, United States

**Keywords:** sinoatrial node, adenylyl cyclase, heart rate, heart rate variability, adenylyl cyclase type 8, parasympathetic activity, sympathetic activity, ivabradine

## Abstract

Heart rate (HR) and HR variability (HRV), predictors of over-all organism health, are widely believed to be driven by autonomic input to the sinoatrial node (SAN), with sympathetic input increasing HR and reducing HRV. However, variability in spontaneous beating intervals in isolated SAN tissue and single SAN cells, devoid of autonomic neural input, suggests that clocks intrinsic to SAN cells may also contribute to HR and HRV *in vivo*. We assessed contributions of both intrinsic and autonomic neuronal input mechanisms of SAN cell function on HR and HRV via *in vivo*, telemetric EKG recordings. This was done in both wild type (WT) mice, and those in which adenylyl cyclase type 8 (ADCY8), a main driver of intrinsic cAMP-PKA-Ca^2+^ mediated pacemaker function, was overexpressed exclusively in the heart (TG^AC8^). We hypothesized that TG^AC8^ mice would: (1) manifest a more coherent pattern of HRV *in vivo*, i.e., a reduced HRV driven by mechanisms intrinsic to SAN cells, and less so to modulation by autonomic input and (2) utilize unique adaptations to limit sympathetic input to a heart with high levels of intrinsic cAMP-Ca^2+^ signaling. Increased adenylyl cyclase (AC) activity in TG^AC8^ SAN tissue was accompanied by a marked increase in HR and a concurrent marked reduction in HRV, both in the absence or presence of dual autonomic blockade. The marked increase in intrinsic HR and coherence of HRV in TG^AC8^ mice occurred in the context of: (1) reduced HR and HRV responses to β-adrenergic receptor (β-AR) stimulation; (2) increased transcription of genes and expression of proteins [β-Arrestin, G Protein-Coupled Receptor Kinase 5 (GRK5) and Clathrin Adaptor Protein (Dab2)] that desensitize β-AR signaling within SAN tissue, (3) reduced transcripts or protein levels of enzymes [dopamine beta-hydorxylase (DBH) and phenylethanolamine *N*-methyltransferase (PNMT)] required for catecholamine production in intrinsic cardiac adrenergic cells, and (4) substantially reduced plasma catecholamine levels. Thus, mechanisms driven by cAMP-PKA-Ca^2+^ signaling intrinsic to SAN cells underlie the marked coherence of TG^AC8^ mice HRV. Adaptations to limit additional activation of AC signaling, via decreased neuronal sympathetic input, are utilized to ensure the hearts survival and prevent Ca^2+^ overload.

## Introduction

Heart rate variability (HRV) is a series of complex rhythms buried within beat-to-beat R wave interval time series. HRV is regulated by alterations in autonomic neurotransmitter input from the brain to the sinoatrial node (SAN), and the responses of SAN cells to this input (Yaniv et al., 2014a). Neuronal input modulates an intrinsic coupled-clock system that governs SAN cells automaticity. We have discovered that an intrinsic coupled-clock system [i.e., in the absence of β-adrenergic receptor (β-AR) stimulation] within the SAN cells is crucially dependent on activation of a neuronal-type adenylyl cyclase (AC) type 8 (AC8) that drives cAMP-PKA-Ca^2+^. This cAMP driven signaling is regulated by phosphodiesterase activity to maintain basal pacemaker function near its dynamic mid-range. β-AR stimulation of SAN cells activates intracellular AC signaling, increasing the mean spontaneous action potential (AP) firing rate and reducing inter AP cycle variability (Yaniv et al., 2014a). In contrast, blocking intrinsic AC activity, or its downstream cAMP-dependent signaling, reduces the mean SAN cell AP firing rate and increases intra-AP cycle variability (Vinogradova et al., 2006; Mangoni and Nargeot, 2008 for review; Yaniv et al., 2014a).

Complexity or coherency within the heart rhythm can be estimated from EKG RR time series. Complexity is largely driven by muscarinic cholinergic input to the SAN, while coherency results largely from sympathetic autonomic input to the SAN (Goldberger, 1991; Goldberger et al., 2002; Thayer et al., 2010). Based upon our findings that a coupled-clock system regulates the spontaneous AP firing rate of isolated SAN cells (Lakatta et al., 2010; Yaniv et al., 2014b), we hypothesized that overexpression of AC8 within the SAN cells would generate an increased mean HR in TG^AC8^ mice *in vivo*. We speculated that this would be due to mechanisms intrinsic to SAN cells and would be accompanied by a markedly coherent heart rhythm *in vivo* (McCraty and Zayas, 2014; Smith et al., 2017), rather than to an increased sympathetic input into the SAN. The coherent rhythm would manifest as a reduced variability of EKG RR intervals in the time domain, and a marked reduction in both total power and other rhythm components in the frequency domain in the presence or absence of autonomic blockade. We also reasoned that in the context of HR and HRV changes induced by AC8 overexpression, we would see unique adaptations to limit extrinsic adrenergic input to the SAN, defending against Ca^2+^ overload and ensuring heart survival (Koch et al., 2000).

To test this, we utilized a transgenic mouse (Lipskaia et al., 2000) in which AC8 was exclusively overexpressed in the heart by putting it under control of the myosin heavy chain promotor. We performed comprehensive HR and HRV analyses of EKG recordings from surgically implanted telemeters in unrestrained, untethered TG^AC8^ and their wild type (WT) littermates in the presence of single or dual autonomic receptor blockade. In order to understand how extrinsic autonomic input impacted HR and HRV in TG^AC8^, we (1) gathered EKG recordings in the presence of single or dual sympathetic and cholinergic autonomic receptor blockades; (2) assessed the HR response to a β-adrenergic agonist; (3) measured transcripts of genes and expression of proteins that regulate β-AR sensitivity and catecholamine synthesis in SAN tissue; and (4) measured circulating plasma catecholamine levels.

## Materials and Methods

### Animals

All studies were performed in accordance with the Guide for the Care and Use of Laboratory Animals published by the National Institutes of Health (NIH Publication no. 85-23, revised 1996). The experimental protocols were approved by the Animal Care and Use Committee of the National Institutes of Health (protocol #441-LCS-2016). A breeder pair of TG^AC8^ mice, generated by ligating the murine α-myosin heavy chain promoter to a cDNA coding for human AC8 (Lipskaia et al., 2000), were a gift from Nicole Defer/Jacques Hanoune, Unite de Recherches, INSERM U-99, Hôpital Henri Mondor, F-94010 Créteil, France. WT littermates, bred from the C57/BL6 background, were used as controls.

### Telemetry

Telemetry sensors (ETA-F20 or HDX-11, Data Sciences International, St. Paul, MN, United States) were surgically implanted into WT and TG^AC8^ mice under 2% isoflurane anesthesia administered by nosecone. Mice were allowed to recover for 2 weeks before any recordings were performed (Thireau et al., 2008). First, 24-h EKGs were recorded during a normal light–dark cycle using RPC-1 receiver plates with a sampling rate of 1000 Hz. All other data were analyzed during the mouse sleep cycle, wherein a 90-min baseline recording was obtained and then intraperitoneal injections of a saline solution containing the drug of interest were administered. This was followed by an additional 90 min of EKG recording. The injection volume was 200 μL/30 g mouse, wherein atropine (0.5 mg/kg), or propranolol (1 mg/kg) alone, or together were administered. In other mice, the β-AR agonist, dobutamine (5 mg/kg), was tested individually, followed 60 min later by the administration of combined atropine (0.5 mg/kg) and propranolol (1 mg/kg). All recordings were performed 48 h apart. Representative EKG recordings are illustrated in Figure 2A.

### Average Heart Rate (RR Interval) and Heart Rate Variability (RR Interval Variability) Analyses

Heart rate variability analyses were performed using a combination of LabChart 7.37 and custom python 3.5 software. EKGs were first analyzed to identify segments that fit strict selection criteria. This was mainly dependent on whether the HR was stationary and devoid of ectopic beats. Ectopic beats were defined as any interval occurring outside two standard deviations of the mean. The determination of stationarity was based on the absence of linear trends, or a stable HR. Segments that contained greater than 2048 accurate intervals, as assessed directly by hand, were then run through the in-house software developed in python 3. The data were cleaned by removing outliers greater than two standard deviations, i.e., ectopic beats, for time domain and non-linear analyses and replacing ectopic beats with a 0 for frequency-based analyses. For each animal, a set of 2048 RR intervals were transformed using a fast Fourier transform. The absolute values of the data were then squared and divided by the average interval length to give a usable transformed dataset. The set was then further broken down into ranges, in which high frequency power constituted values between 1.5 and 5.0, low frequency power between 0.4 and 1.5, and very low frequency between 0 and 0.4. Poincaré SD values were calculated by adding (for SD1) or subtracting (for SD2) successive RR intervals and then taking the square root of the variance of the set divided by the square root of 2. Multiscale entropy was calculated using the PhysioZoo algorithm for SampEn, modified to only output E1 in python 3 (Behar et al., 2018).

### Sinoatrial Node (SAN) and SAN Cell Isolation

Mice were injected (intraperitoneally) with heparin and acutely anesthetized with pentobarbital-based euthanasia solution. The heart then was quickly removed and placed into Tyrode solution containing (mM): 140.0 NaCl, 10.0 HEPES, 10.0 glucose, 5.4 KCl, 1.2 KH_2_PO_4_, 1.8 CaCl_2_, and 1.0 MgCl_2_; pH was adjusted to 7.4 with NaOH. The SAN region was identified anatomically, under a dissecting microscope, between inferior and superior vena cava, crista terminalis, and intra-atrial septum and cut into strips perpendicular to the crista terminalis, washed (three times) for 5 min at 35°C in low Ca^2+^ Tyrode solution containing (mM): 140.0 NaCl, 10.0 HEPES, 20.0 glucose, 0.06 CaCl_2_, 5.4 KCl, 1.2 KH_2_PO_4_, 50 taurine, and 1 mg/1 mL bovine serum albumin (BSA), pH 6.9 with NaOH. Next, SAN strips were incubated for 30 min in the same solution with addition of collagenase type 2, protease type XIV, and elastase at 35°C. After enzymatic digestion, tissue was washed (x3) in modified high potassium (KB) solution containing (mM): 100 potassium glutamate, 10 potassium aspartate, 10 Hepes, 20 glucose, 25 KCl, 2 MgCl_2_, 10 KH_2_PO_4_, 20 taurine, 5 creatine, 0.5 EGTA, 1 mg/1 mL BSA, and 5.0 β-hydroxybutyric acid, pH 7.2 with KOH, and kept at 4°C for 1 h with 50 mg/mL polyvinylpyrrolidone (PVP). Finally, cells were dispersed by gentle pipetting in the KB solution and stored at 4°C. For immunolabeling studies, 250 μL of freshly isolated SAN cells in KB solution was added to each laminin coated glass (0 size) bottom MatTek dish (35 mm).

### RNA Extraction, cDNA Synthesis, RT-qPCR, and Data Analysis

RT-qPCR of SAN tissue was performed to determine the transcript abundance of human AC8 genes that mediate neural autonomic input to SAN cells and for genes that regulate cardiac catecholamine synthesis (Wang et al., 2017). RNA was extracted from isolated mouse SAN (*n* = 3 pooled samples from TG^AC8^ and WT, each sample collected from four mice) or left ventricular myocytes (VMs) (*n* = 4 WT and 4 TG^AC8^ mice) with RNeasy Mini Kit (Qiagen, Valencia, CA, United States) and DNAse on column digestion; 2 μg of total RNA was used for cDNA synthesis with MMLV reverse transcriptase (Promega) in final 50 μL volume. Primers used for each transcript assessed are listed in Supplementary Table S4. RT-qPCR was performed on QuantStudio 6 Flex Real-Time PCR System (Thermo Fisher Scientific) with 384-well platform. Reaction was performed with FastStart Universal SYBR Green Master Kit with Rox (Roche) using manufacturer recommended conditions, and dissociation curve acquisition; all appropriate controls were included (no template; no RT control). Preliminary reactions were performed for determination of efficiency of amplification and primers validation. Each well contained 0.5 μL of cDNA solution and 10 μL of reaction mixture. Each sample was quadruplicated and repeated twice using *de novo* synthesized cDNA sets. RT-qPCR analysis was performed using ddCt method. Expression level of transcripts was normalized on expression of HPRT level.

### Adenylyl Cyclase Activity

After isolation, mice SAN tissue was frozen in liquid nitrogen and homogenized in 1.5 mL plastic tube with Bel-Art^TM^ SP Scienceware^TM^ liquid nitrogen-cooled Mini Mortar. SAN tissue powder was further lysed in the lysis buffer containing: 70 mM Tris, pH 7.6, 0.5 mM DTT, 1 mM EGTA, 5 mM MgCl2, 0.2 mM IBMX, and 0.33% PIC, sonicated on ice (three pulses, 15 s each) and further rotated on ice for 15 min. Then lysate was centrifuged during 10 min at 1000 × *g* at 4°C to remove debris. Supernatant was used to measure protein content and to detect AC activity in the lysate. AC reaction composition: 70 mM Tris (pH 7.6), 0.5 mM DTT, 1 mM EGTA, 5 mM MgCl2, 0.2 mM IBMX, 0.25% PIC, 1 mM ATP, 5 mM creatine phosphate, 60 U/mL creatine phosphokinase, 0.2% DMSO, and a sample (SAN lysate supernatant, 100 μg of total protein in 100 μL of AC reaction). AC reaction lasted for 5 min at 35°C and was stopped by boiling during 5 min. Later AC reaction solution was cooled down, centrifuged for 15 min at 4°C at 20,000 × *g* and the supernatant was used for cAMP measurement; 9 μL of the supernatant was used in the LANCE assay (Lance cAMP384 kit 500 points, Perkin Elmer, AD0262) in a total volume of 24 μL. Buffer for the LANCE assay standard curve was prepared exactly as samples including boiling and centrifugation steps. All measurements were done in triplicate. Protein concentration in samples was detected by Reducing Agent Compatible Pierce^®^ Microplate BCA Protein Assay Kit # 23252.

### Immunolabeling of Single Isolated Sinoatrial Node Cells

Immunolabeling of selected proteins expressed by genes examined by RT-qPCR was performed in freshly isolated mouse SAN cells. Cells were plated on laminin coated MatTek dishes for 1 h. For immunofluorescence staining, cells were fixed with 4% paraformaldehyde for 15 min at room temperature, washed three times with PBS, and then permeabilized with 0.2% Triton X-100 in PBS for 10 min at room temperature. The plates were washed two more times with PBS and then incubated with 10% goat serum for 1 h to minimize non-specific staining. Afterward, samples were incubated at 4°C overnight with primary antibodies against RGS6 (EPR6342) ab128943, DAB 2 (10109-2-AP), GRK 5 (ab64943), TH (ab112), HCN4 (MBS800358), and Adcy8 (bs-3925R) all in dilution 1:100. Cells were then washed three times with PBS and incubated with fluorescence-conjugated secondary antibodies (1:1000) (Sigma, United States) for 45 min at 37°C. Cell nuclei were labeled with DAPI (Sigma, United States). Cells were visualized by LSM 710 laser-scanning confocal microscope (Carl Zeiss) and images were captured using the Carl Zeiss Zen software. Quantitative fluorescence image analysis was performed with Image J software, according to the following protocol: http://theolb.readthedocs.io/en/latest/imaging/measuring-cell-fluorescence-using-imagej.html. Images of stained cells were transferred and analyzed with ImageJ software to calculate the basic measurements of each image, including area, mean gray value, and integrated density. To calculate the corrected total cell fluorescence (CTCF)^*^, small areas of positively stained fluorescent cells were selected using free hand selection tool. A background reading was created by selecting a negatively stained rectangular section near the analyzed cell. From the results, total fluorescence per cell was calculated in Excel with the following formula:

^*^CTCF = integrated density – (area of selected cell × mean fluorescence of background readings).

### Plasma Catecholamine Measurements

Plasma concentrations of catecholamines (noradrenaline, adrenaline, Dopa, and dopamine) and their degradation products: 3,4-dihydroxyphenylglycol (DHPG) and 3,4-dihydroxy-phenylacetic acid (DOPAC) were quantified by high performance liquid chromatography with electrochemical detection. Concentrations of catecholamines were determined after extraction from plasma using alumina adsorption according to previously described methods (Eisenhofer et al., 1986).

### Statistical Analyses

Statistical analyses of EKG data employed RStudio (RStudio Team, 2017) and R 3.2.3 (R Core Development Team, 2017). A linear mixed effects model (lmerTest; Kuznetsova et al., 2016) was used to compare EKG data from WT littermates and TG^AC8^, prior to and following drug administration, looking at both drug effects, genotype effects, and drug–genotype interactions, i.e., different responses to drugs in WT and TG^AC8^ mice. For each comparison, the basal for that day was used to compare the drug effect for that day, i.e., there was a separate analysis done for each pre- and post-drug data gathered. For individual comparisons, the differences of least squares means were calculated with Satterthwaite approximation for degrees of freedom (lmerTest; Kuznetsova et al., 2016). Drug responses of HR and HRV were compared to pre-drug controls in each mouse. For the comparison of average HR over 24 h, a one-way ANOVA was used to detect a genotypic difference. RT-qPCR, immunolabeling, and plasma catecholamine data were analyzed by Student’s *t*-test (*p*-value < 0.05 was taken as statistically significant).

## Results

### AC8 Transcripts and Protein Expression Are Increased in TG^AC8^ SAN

**FIGURE 1:**
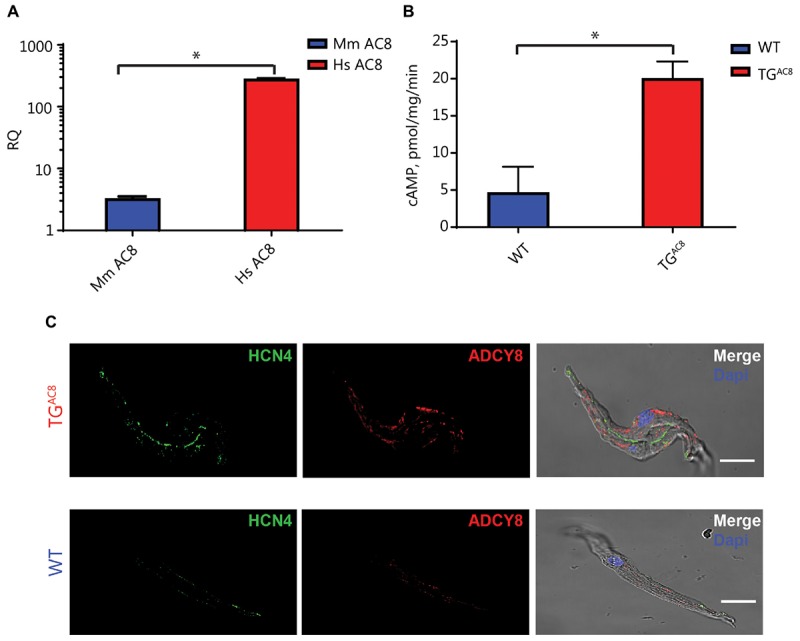
Transcript abundance and immunolabeling of ADCY8 and AC activity in SAN tissue. **(A)** Relative quantification (RQ) transcript abundance of mouse (Mm) vs. human (Hs) AC8 in TG^AC8^ SAN tissue (*n* = 3, ^∗^*p* < 0.0001). **(B)** AC activity of SAN tissue lysates is increased in TG^AC8^ vs. WT mice (*n* = 4, ^∗^*P* < 0.01). **(C)** AC protein expression detected by immunolabeling for ADCY8 is increased in TG^AC8^ vs. WT mice. Left panels: HCN4 immunolabeling; middle panel: selected antibody immunolabeling; right panel: overlay of left and center panels. Scale bar, 20 μm.

We first showed that AC8 transcripts are expressed in SAN. Figure 1A demonstrates that a high level of human AC8 transcript was expressed in TG^AC8^ SAN tissue (Figure 1A, amplification plots of RT q-PCR are provided in Supplementary Figure S1). The expression of mouse AC8 transcripts in TG^AC8^ was markedly decreased compared to human. AC8 protein expression, assessed by immunostaining of single isolated SAN cells, was also increased in TG^AC8^ vs. WT (Figure 1C and Supplementary Table S1). Further, SAN tissue AC activity was markedly increased in TG^AC8^ vs. WT (Figure 1B).

### Basal HR

**FIGURE 2:**
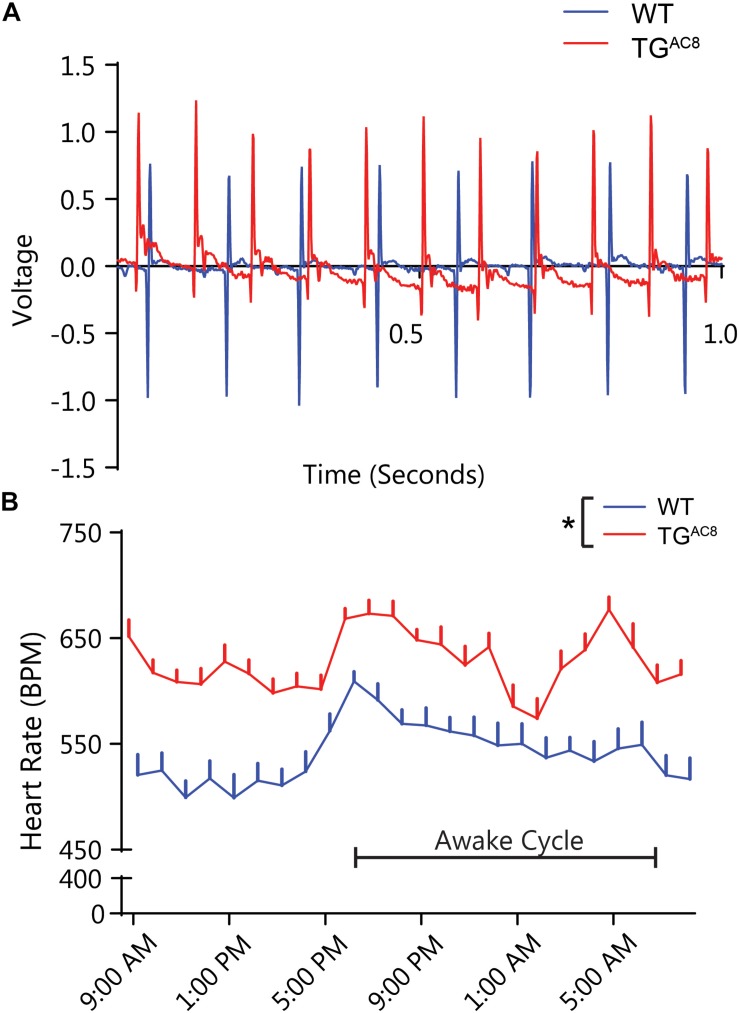
*In vivo* telemetric EKG recordings in awake, unrestrained TG^AC8^, and WT mice over a 24-h period. **(A)** Representative tracings of TG^AC8^ and WT mice EKG in the basal state. **(B)** Sixty-minute averages of basal HR were elevated in TG^AC8^ compared to WT throughout the 24-h period. *N* = 6 for WT and *N* = 7 for TG^AC8^. ^*^p < 0.05 for differences determined by a one-way ANOVA.

Figure 2A illustrates representative telemetric EKG recordings from a TG^AC8^ and WT mouse. Analysis of 60-min averages of 24-h EKG telemetric recordings demonstrates that the average HR of TG^AC8^ mice is higher than that in WT over the entire 24-h period (Figure 2B), providing evidence to support the idea that AC activity intrinsic to SAN cells is a determinant of HR *in vivo*.

### RR Interval Variability Are Markedly Reduced in TG^AC8^

**FIGURE 3:**
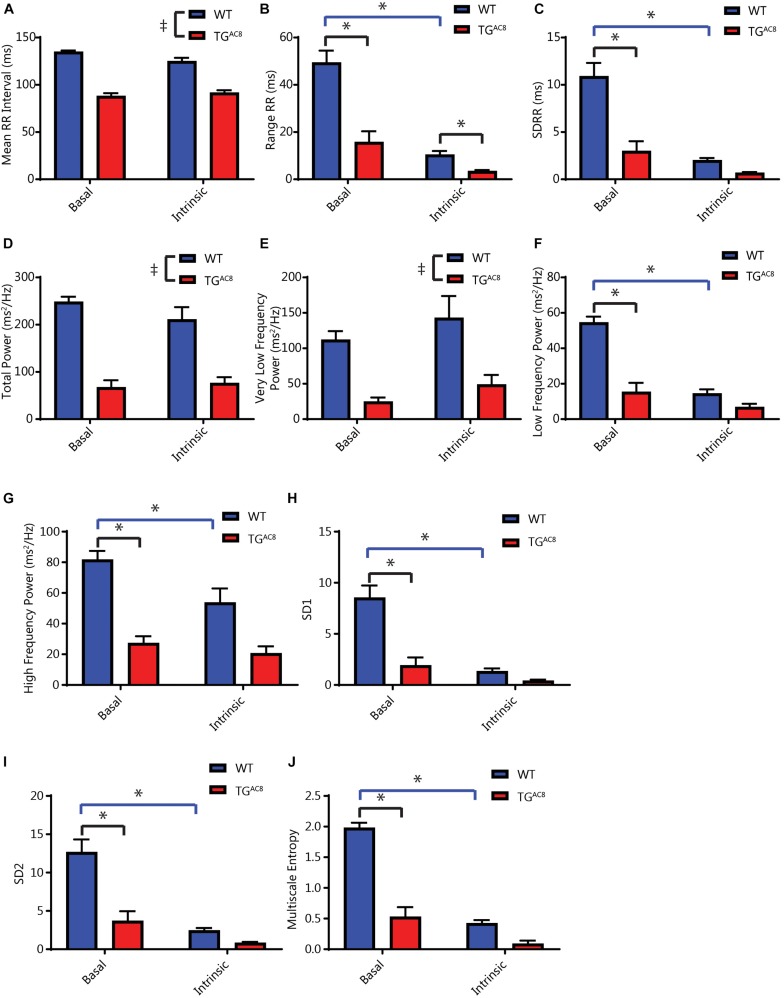
Analyses of average basal and intrinsic (dual autonomic blockade) HR and selected HRV parameters of *in vivo* EKG time series. **(A)** Average RR interval length. **(B)** Range of RR intervals, interaction *F*-value of 30.223, *p*-value of 0.0002. **(C)** Standard deviation of RR intervals, interaction *F*-value of 20.541, *p*-value of 0.001. **(D)** Total power. **(E)** Very low frequency power. **(F)** Low frequency power, interaction *F*-value of 14.415, *p*-value of 0.003. **(G)** High frequency power, interaction *F*-value of 5.184, *p*-value of 0.046. **(H)** Poincare plot SD1, interaction *F*-value of 24.921, *p*-value of 0.0005. **(I)** Poincare plot SD2, interaction *F*-value of 17.884, *p*-value of 0.001. **(J)** Multiscale entropy, interaction *F*-value of 119.116, *p*-value of 7.09E-07. ^T^*p* < 0.05 for main effects of drug differences. ^*^p < 0.05 for significant pairwise differences, if a significant interaction effect was found, as determined by a linear mixed effects model. *F*-value and *p*-value provided for significant interactions. *N* = 6 for WT and *N* = 6 for TG^AC8^.

The constitutively increased HR (Figure 2B) is accompanied by a marked reduction in both the mean RR and the range of RR intervals measured in the basal state (Figures 3A,B). Selected RR variability measures are illustrated in Figures 3B–J. A comprehensive statistical analysis of all measured RR interval variability parameters is provided in Supplementary Table S2. Basal RR variability in the time domain, i.e., standard deviation of RR intervals (SDRR) is two- to threefold lower in TG^AC8^ than WT (Figure 3C). Rhythms that range over frequencies less than half of the mean HR are detected in the frequency domain, i.e., within the power spectrum derived fast Fourier transforms. A representative power spectrum for WT and TG^AC8^ is shown in Supplementary Figure S2. Basal RR interval total power and power in very low, low, and high (frequency domains) are also two- to threefold lower in TG^AC8^ than WT (Figures 3D–G). Non-linearity of rhythms buried within an EKG time series, reflected by SD1, SD2, and multiscale entropy of RR intervals, was also markedly decreased in TG^AC8^ vs. those in WT (Figures 3H–J). Taken together, the results in Figure 3 demonstrate a low mean basal RR interval (high basal HR) in TG^AC8^ is accompanied by a markedly coherent heart rhythm that underlies reduced RR variability in both time and frequency domains.

### Average RR Intervals and RR Interval Variability in the Presence of Double Autonomic Blockade

We repeated the HR and HRV in measurements in the presence of dual autonomic blockade (atropine and propranolol) in order to gauge the relative effects of autonomic input on intrinsic HR and HRV in both genotypes. Dual autonomic blockade had substantially larger effects on many HRV descriptors in WT than TG^AC8^ (Figures 3A–J), providing an initial inference for genotype differences in neurotransmitter input. Importantly, the reduction in mean basal RR and the concomitant coherent pattern of RR variability persisted in the presence of dual autonomic blockade for multiple HRV parameters (Figures 3A,B,D,E). This suggests that HR and coherent HRV patterns of TG^AC8^ are dominated by mechanisms intrinsic to AC8 overexpressed SAN cells.

### Response to Single Autonomic Receptor Blockade

**FIGURE 4:**
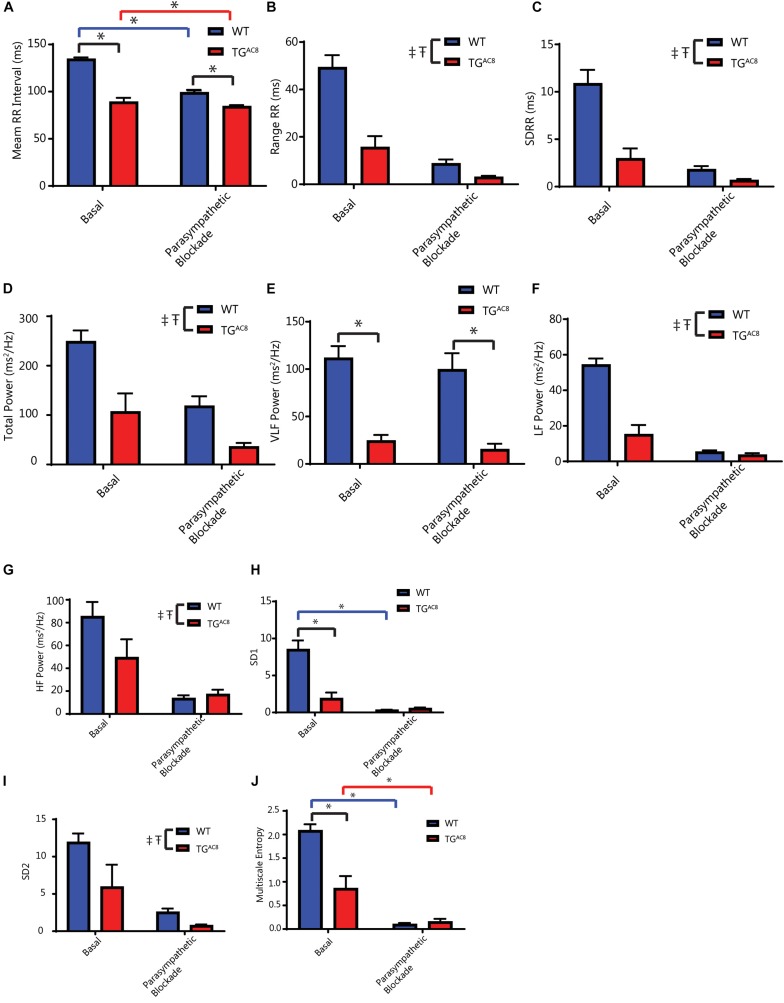
Effects of parasympathetic blockade (atropine) on mean *in vivo* RR intervals and selected HRV parameters. **(A)** Average RR interval, interaction *F*-value of 10.883, *p*-value of 0.009. **(B)** Range of RR intervals. **(C)** Standard deviation of RR intervals. **(D)** Total power. **(E)** Very low frequency power. **(F)** Low frequency power. **(G)** High frequency power. **(H)** Poincare plot SD1, interaction *F*-value of 7.367, *p*-value of 0.023. **(I)** Poincare plot SD2. **(J)** Multiscale entropy (E1), interaction *F*-value of 26.180, *p*-value of 0.0006. ^‡^*p* < 0.05 for main effects of genotype differences. T*p* < 0.05 for main effects of drug differences. ^*^p < 0.05 for significant pairwise differences, if a significant interaction effect was found, as determined by a linear mixed effects model. *F*-value and *p*-value provided for significant interactions. *N* = 6 for WT basal and *N* = 5 for TG^AC8^.

Because the marked reduction in mean RR and RR variability was not only present in the intrinsic state, but also in the basal resting state, we assessed the effects of single autonomic blockade on HR and HRV in TG^AC8^ and WT (Figure 4 and Supplementary Table S2).

The cholinergic receptor blocker, atropine, reduced the mean basal RR interval in WT to a far greater extent than in TG^AC8^ (Figure 4A). Atropine also reduced time domain basal RR interval range in both genotypes (Figure 4B). Atropine reduced both the basal SDRR (Figure 4C) and total power frequency domain RR variability (Figures 4D,F,G) in both genotypes. Atropine significantly reduced non-linear basal RR interval variability parameters to a greater extent in WT than in TG^AC8^ (Figures 4H,J). Taken together these results indicate that blockade of basal state parasympathetic receptor signaling reduces the genotype differences in a “para-sympathetic” like neuronal input. This suggests that in the absence of atropine, responses of SAN to parasympathetic input are substantially reduced in TG^AC8^ vs. WT.

There were some genotypic differences in HR and HRV in response to propranolol, a sympathetic receptor blocker (Supplementary Table S2). Time domain and non-linear measures of RR intervals, i.e., SDRR, CV, MSE, etc., show a difference following sympathetic blockade, but measures of frequency domain failed to display altered genotypic effects. This may indicate that the autonomic sympathetic signaling to the SAN or the response to activation of sympathetic autonomic receptors is altered in the TG^AC8^ mice to accommodate the increased intrinsic sympathetic-like signaling.

### Reduced Effectiveness of Extrinsic Adrenergic Input Into SAN

**FIGURE 5:**
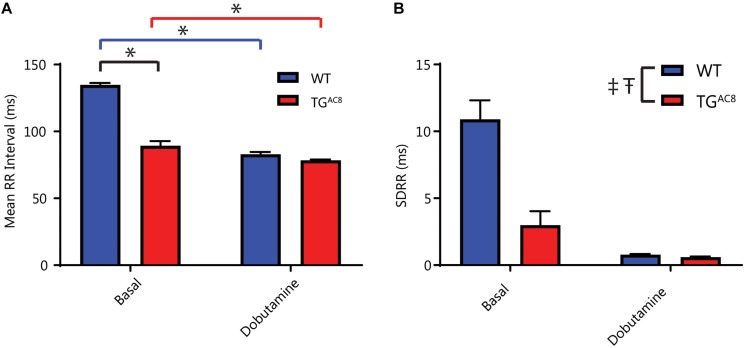
The effects of β-AR stimulation *in vivo*. **(A)** Effect of the β1-AR agonist, dobutamine on mean RR, interaction *F*-value of 14.156, *p*-value of 0.003. **(B)** The SDRR in response to dobutamine. ^‡^*p* < 0.05 for main effects of genotype differences. ^T^*p* < 0.05 for main effects of drug differences. ^*^p < 0.05 for significant pairwise differences, if a significant interaction effect was found, as determined by a linear mixed effects model. *F*-value and *p*-value provided for significant interactions. *N* = 6 for WT basal and *N* = 6 for TG^AC8^.

Because the increased HR and coherence within TG^AC8^ results from high levels of intrinsic cAMP-PKA-Ca^2+^ signaling, we reasoned that adaptive mechanisms within TG^AC8^ are likely utilized to limit the additional activation of cAMP-PKA-Ca^2+^ dependent catecholamine signaling. In order to test this hypothesis, we sought to utilize a dominant external adrenergic input to SAN cells via activation of β-ARs by adrenergic neurotransmitters. We stimulated β-ARs via infusion of dobutamine, a β_1_-AR agonist, in TG^AC8^ and WT mice. The administration of dobutamine produced a potent effect to reduce the mean RR interval in WT mice, but its effect to reduce the mean RR in TG^AC8^ mice was markedly reduced (Figure 5A). Dobutamine produced a reduction in RR interval in TG^AC8^ and WT mice, with the magnitude of the effect being larger in WT mice (Figure 5B). Because β-AR stimulation increases intracellular cAMP-PKA-Ca^2+^ signaling in SAN cells (Vinogradova et al., 2006), the effects of dobutamine infusion should mimic overexpression of AC8 in the SAN cells of WT animals. In other terms, in the presence of β-AR stimulation, mean HR in WT mice resembles the mean HR patterns prior to dobutamine in TG^AC8^ mice.

**FIGURE 6:**
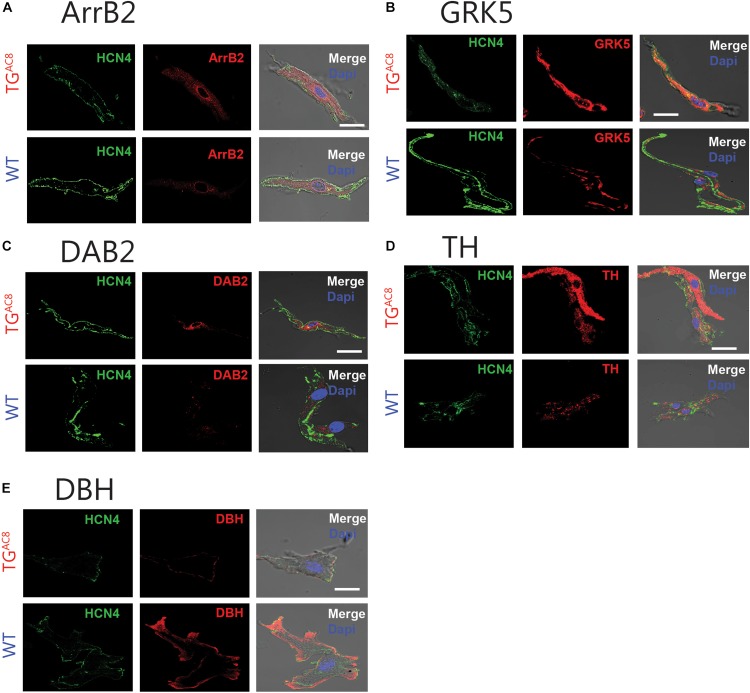
Representative example of immunostaining of single, isolated SAN cells for proteins involved in β-AR desensitization and catecholamine synthesis in WT and TG^AC8^ for **(A)** Arrb2 and **(B)** GRK5. **(C)** DAB2. **(D)** Protein levels of TH and **(E)** DBH, enzymes involved in catecholamine synthesis in intrinsic cardiac adrenergic cells. Left panels: HCN4 immunolabeling; middle panel: selected antibody immunolabeling; right panel: overlay of left and center panels. Scale bar, 20 μm.

One plausible mechanism for the reduced responsiveness of the TG^AC8^ heart to β-adrenergic neurotransmitters is through desensitization of β-Ars (Koch et al., 2000). We assessed the expression of selected markers of β-AR desensitization in TG^AC8^ and WT SAN tissue and single SAN cells. Transcripts for β-Arrestin (Arrb_2_), G Protein-Coupled Receptor Kinase 5 (GRK5) (BARK), and Dab2 were elevated in TG^AC8^ vs. WT (Supplementary Table S3). This was further confirmed by immunolabeling of single, isolated, HCN4 positive SAN (Figure 6 and Supplementary Table S1). HCN immunolabeling did not differ in TG_AC8_ vs. WT SAN cells (Supplementary Table S3). Neither transcripts of genes coding for β-ARs (ADRB1, ADRB2, ADRB3), nor for selected G proteins [Guanin Nucleotide binding protein, alpha stimulating (GNAS), G Protein Subunit Alpha I2 (GNAI2), G Protein Subunit Alpha I3 (GNAI3)] differed in TG^AC8^ compared to WT (Supplementary Table S3). Interestingly, transcript abundance of RGS2, which inhibits Gi signaling, leading to an inactivation of AC in SAN cells (Yang et al., 2012), was also reduced. There also was a trend toward a reduction in Regulator of G Protein Signaling 2 (RGS2) and Regulator of G Protein Signaling 6 (RGS6) (Yang et al., 2010) in TG^AC8^ vs. WT (Supplementary Table S3), and a significant reduction in RGS6 protein (Supplementary Table S1).

### Enzymes Involved in Cardiac Catecholamine Production Are Altered in TG^AC8^ SAN Tissue and Cells

We postulated that, in addition to desensitization of β-AR stimulation to catecholamines, the production of catecholamines that stimulates these receptors may also be reduced in TG^AC8^ as part of a strategy to block external adrenergic input. Catecholamines that stimulate SAN cells β-ARs can arise from multiple sources, including autonomic nerve endings within the heart, from circulating plasma or from intrinsic cardiac adrenergic cells. Intrinsic cardiac adrenergic cells are populated in different locations within the murine heart including the SAN, which express enzymes that synthesize catecholamines from tyrosine (Huang et al., 1996; Wang et al., 2017).

We measured mRNA transcripts in SAN tissue (Supplementary Table S3) and protein expression in single isolated SAN cells (Supplementary Table S1) for enzymes that effect the conversion of tyrosine to L-DOPA (tyrosine hydroxylase, TH); L-DOPA to dopamine (DOPA decarboxylase); dopamine to norepinephrine [dopamine beta-hydorxylase (DBH)]; and norepinephrine to epinephrine [phenylethanolamine *N*-methyltransferase (PNMT)]. Compared to WT, protein levels for TH were increased in TG^AC8^ HCN4 positive SAN cells, while DBH protein was reduced (Figure 6 and Supplementary Table S1). PNMT transcripts were also reduced in TG^AC8^ vs. WT SAN tissue (Supplementary Table S3).

### Circulating Plasma Catecholamine Levels Are Altered in TG^AC8^

**FIGURE 7:**
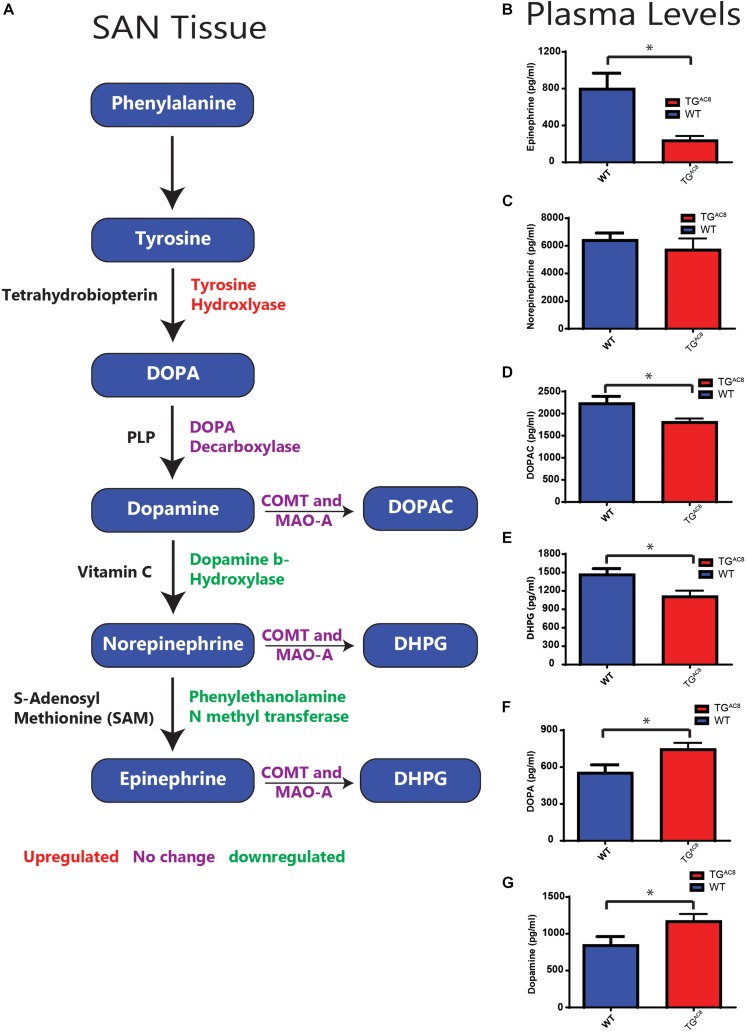
A schematic of enzymes involving catecholamine synthesis and degradation in SAN tissue (cf. Supplementary Tables S1, S3 for quantitative analysis) and circulating plasma catecholamines in TG^AC8^ and WT mice. **(A)** Red indicates genes upregulated, purple no change, and green downregulated in transcript abundance or protein expression in TG^AC8^ vs. WT SAN tissue or isolated SAN cells (cf. Supplementary Tables S1, S3). **(B–G)** Quantification of plasma catecholamine concentrations in TG^AC8^ and WT mice. **(B)** Epinephrine. **(C)** Norepinephrine. **(D)** DOPAC. **(E)** DHPG. **(F)** DOPA. **(G)** Dopamine (*N* = 10 WT and *N* = 10 TG^AC8^). ^*^p < 0.05 for concentration differences determined by Student’s *t*-test.

Although AC8 overexpression in TG^AC8^ mice is cardiac specific (Lipskaia et al., 2000), afferent neuronal signals arising from within the heart can influence autonomic balance (Armour, 2008). As such, we gathered to test plasma catecholamines differences in TG^AC8^ and WT in the context of a confined pathway (Figure 7A). We found that circulating plasma epinephrine was significantly lower in TG^AC8^ vs. WT, and plasma norepinephrine tended to be lower in the TG^AC8^ vs. WT (Figures 7B,C). DOPAC, which is derived from dopamine, and DPHG, which is derived from norepinephrine, were also reduced in TG^AC8^ plasma (Figures 7D,E). In contrast, both DOPA and dopamine levels are increased in TG^AC8^ vs. WT (Figures 7F,G). The altered pattern of circulating plasma catecholamines in TG^AC8^ (Figures 7B–G) mirrors patterns of enzyme expression that regulate catecholamine production in TG^AC8^ SAN tissue (Figure 7A).

## Discussion

### Mechanisms Intrinsic to SAN Cells Rather Than Extrinsic Autonomic Input to the SAN Drive Heart Rate Variability of the TG^AC8^

The rate and beat to beat variability of spontaneous AP generation by the SAN, the central pacemaker of the heart, are highly dependent on characteristics of neurotransmitter signals. This includes the quantity of autonomic neurotransmitter released from nerve endings, its binding to autonomic receptors on SAN cell, transmembrane and intracellular transduction of the signals in response to neurotransmitter activation of β-adrenergic and muscarinic cholinergic receptors to various cellular secondary messengers (such as cAMP, Ca^2+^, and protein phosphorylation). The culmination of neurotransmitter signaling, however, modulation of critical intrinsic SAN cell protein effectors that regulate their automaticity, even in the absence of autonomic input, i.e., the intrinsic automaticity of these cells, which persists in the absence of autonomic neuronal input when these cells are isolated from the SAN (Vinogradova et al., 2006, 2008; Younes et al., 2008; Lakatta et al., 2010).

It has been argued in many previous studies that changes in HRV largely result from alterations in autonomic neural input signals to the SAN (Lahiri et al., 2008; Thayer et al., 2010; Billman, 2011; Malik et al., 2019). There is, however, substantial support for the idea that like HR, regulation of HRV *in vivo* is not solely attributable to autonomic input, but also to mechanisms intrinsic to SAN cells (Boyett et al., 2019) is present *in vivo* during autonomic blockade. Significant beating rate variability has been described via *ex vivo* cardiac preparations. In the absence of external autonomic input, i.e., in isolated, adult hearts, isolated SAN tissue and isolated SAN cells (Rocchetti et al., 2000; Zaza and Lombardi, 2001; Zahanich et al., 2011; Monfredi et al., 2013, 2014; Yaniv et al., 2014a, b; Tsutsui et al., 2018), as well as in human embryonic cells (Mandel et al., 2012), and induced pluripotent stem cell-derived cardiomyocytes (Ben-Ari et al., 2014).

Intrinsic mechanisms that drive the spontaneous beating rate and rhythm in isolated SAN cells are embodied within a coupled-clock system driven by Ca^2+^ calmodulin activated AC type 8 (AC8). Stimulation of autonomic receptors of single SAN cells in isolation not only alters their beating rate but also their beat to beat variability (Yaniv et al., 2013, 2014a) via modulation of the same intrinsic pacemaker cell mechanisms that link autonomic neurotransmitter signaling to its effects *in vivo*.

Although *in vivo* autonomic neural input to the SAN must modulate HR and HRV *via* signaling its effects to effector mechanisms intrinsic to the SANC coupled-clock system, whether or not the AC-driven coupled clock system intrinsic to SANC has a role in regulation of HR and HRV *in vivo* had never been clearly established. To address this conundrum, we utilized a transgenic mouse in which a critical component of the human coupled clock system, the human AC8 gene, was genetically overexpressed (TG^AC8^) in a cardiac specific manner (Georget et al., 2002; Lipskaia et al., 2000). We hypothesized that a potent coherent intrinsic “sympathetic-like” state of TG^AC8^ might largely override autonomic input to the SAN, partially uncoupling the SAN from autonomic neural surveillance, thereby unmasking a potent intrinsic SANC component of HR and HRV regulation *in vivo*. In other terms, we hypothesized that the TG^AC8^ heart will manifest an intrinsically coherent HR and HRV that are largely independent of whole body and neurovisceral integration (McCraty and Zayas, 2014; Smith et al., 2017). To this end, we performed comprehensive HR and HRV studies in unrestrained, unanesthetized mice in the absence and presence of single or dual autonomic receptor blockade (atropine and propranolol), and measured plasma catecholamine levels and transcription of genes and expression of proteins that regulate the responses to autonomic receptor input.

Our results show, for the first time, that markedly increased AC8 transcription and translation in TG^AC8^ SAN cells (Figure 1 and Supplementary Table S1) produces marked increases in the mean HR *in vivo* in awake, untethered TG^AC8^ mice (Supplementary Figure S2), and that the *in vivo* HRV shifts to a markedly coherent pattern (Figure 3). Not only was mean basal *in vivo* HR considerably elevated and basal HRV markedly reduced in TG^AC8^ compared to WT, but the mean intrinsic HR (i.e., HR in the presence of dual autonomic blockade) was also elevated in TG^AC8^, while intrinsic HRV was reduced (Figure 3). The *in vivo* HRV pattern exhibited by TG^AC8^ is largely attributable to coupled-clock mechanisms intrinsic to SAN cells that are driven by AC8. In other terms, mechanisms intrinsic to the TG^AC8^ SAN regulate mean HR and HRV. Likewise, the TG^AC8^ heart evades autonomic surveillance to a substantial degree. In this regard, mechanisms within TG^AC8^ SAN cells appear to function as autonomic ganglia. Because mechanisms intrinsic to SAN cells dominate TG^AC8^ HR and HRV *in vivo*, this experimental model provides proof of principle that cAMP-directed signaling intrinsic to SAN cells can markedly impact not only on mean HR but also HRV.

Significant cAMP-mediated beating rate variability, i.e., HRV in the absence of external autonomic input, is attributable to intrinsic coupled-clock mechanisms has already been described via *ex vivo* cardiac preparations. Specifically, beat-to-beat variability occurs in isolated, adult hearts, isolated SAN tissue and isolated SAN cells (Monfredi et al., 2013; Yaniv et al., 2014a), as well as in human embryonic cells (Mandel et al., 2012), and induced pluripotent stem cell-derived cardiomyocytes (Ben-Ari et al., 2014).

### Adaptive Strategies of TG^AC8^ to Blunt Adrenergic Autonomic Input

We discovered that in the context of high cAMP-PKA-Ca^2+^ signaling intrinsic to SAN cells, TG^AC8^ applies adaptive strategies in order to blunt additional external sympathetic input. Adaptations that partially disengage the heart from external adrenergic input are important to prevent arrhythmias, cell necrosis, and apoptosis, thus preventing cardiomyopathy and death of the organism (Koch et al., 2000). Adaptive strategies in TG^AC8^ include (1) downregulation of mechanisms involved in catecholamine synthesis of intrinsic cardiac adrenergic cells within SAN tissue (Huang et al., 1996), (2) reductions in circulating plasma catecholamines, and (3) upregulation of pathways that desensitize β-AR receptor signaling.

### Catecholamine Production in the Heart

Prior studies have demonstrated that intrinsic cardiac adrenergic cells within left ventricular myocardium contribute about 15% of total heart epinephrine, norepinephrine, or dopamine levels (Huang et al., 1996). We provide further evidence that enzymes involved in catecholamine synthesis are expressed in cells that reside within the adult SAN. Protein levels of TH, the initial enzymatic step involved in catecholamine production, was increased in TG^AC8^ vs. WT HCN4-immunolabeled SAN cells (Supplementary Table S1 and Figures 6, 7), suggesting that some pacemaker cells in adult SAN may also be intrinsic cardiac adrenergic cells. These intrinsic cardiac adrenergic cell-derived transmitters stimulate G protein coupled receptors in an autocrine/paracrine manner (Peoples et al., 2018). We assessed protein expression and transcript abundance of DBH and transcripts coding for PNMT, enzymes that catalyze the conversion of dopamine to norepinephrine or norepinephrine to epinephrine. Of note, both DBH and PNMT were reduced in TG^AC8^ vs. WT SAN tissue and cells (Supplementary Table S1 and Figure 7A). Downregulation of DBH and PNMT in TG^AC8^ (Figure 7A) may indicate that intrinsic norepinephrine and epinephrine production by intrinsic cardiac adrenergic in TG^AC8^ SAN cells is less than that of WT. Normal fetal heart development requires DBH expression, as embryonic heart failure and death ensue when the protein coding gene is knocked out (Thomas et al., 1995). The pattern of upregulation of TH in SAN cells coupled to a reduction of DBH may indicate that, as in circulating plasma (Figure 7G), the concentration of dopamine is also increased in SAN tissue. Of note, dopamine can limit cell AC activity via activation of G_1_ coupled muscarinic receptor signaling (Missale et al., 1998). A reduction in norepinephrine and epinephrine synthesis and increased dopamine could be construed to be adaptive mechanisms employed to reduce intrinsic cardiac adrenergic signaling in TG^AC8^ SAN cells, not only to limit the HR in TG^AC8^ from going beyond the elevated values observed, but also to ensure survival of the TG^AC8^ heart (Koch et al., 2000). Unfortunately, the small volume of mouse SAN precluded any possible measurements of SAN tissue catecholamines. Whether norepinephrine and epinephrine levels are reduced in TG^AC8^ SAN tissue, or dopamine receptor signaling is increased in TG^AC8^ SAN cells merits future study.

### Plasma Catecholamines

Because AC8 overexpression in TG^AC8^ mice is limited to the heart, at first glance, it was surprising to find that circulating catecholamine levels differed between WT and TG^AC8^. The pattern of altered plasma catecholamine levels in TG^AC8^ (Figures 7B–G) mirrors experimental changes from the expression pattern of enzymes involved in catecholamine synthesis within TG^AC8^ SAN tissue or cells (Supplementary Table S1 and Figure 7A). Circulating plasma levels of DOPAC, DPHG, and epinephrine were significantly reduced. Norepinephrine trended toward a significant reduction, and the plasma concentration of dopamine, was significantly increased. In this coherent pacemaker context, cells within the TG^AC8^ SAN would appear to act as an autonomic ganglion (Figures 6, 7), generating signals to modulate catecholamine metabolism in these tissues via signaling directly to brain or indirectly to adrenal medulla (in addition to signaling to other parts of the heart). The altered pattern of circulatory catecholamines in TG^AC8^ is consistent with the idea that the TG^AC8^ heart communicates to/with other organs throughout the body via hemodynamic mechanisms, hormonal signaling, or direct afferent signaling from heat to the spinal cord or brain (Armour, 2008). Alternatively, because catecholamine production by the heart may contribute to plasma catecholamine levels (Huang et al., 1996), the reduction in circulatory plasma catecholamines of the TG^AC8^ may also reflect, in part, reduced cardiac catecholamine synthesis within the TG^AC8^ heart.

### Desensitization of β-Adrenergic Receptor Signaling

The blunted *in vivo* response to dobutamine in TG^AC8^ mice (Figure 5) reflects reduced effectiveness of external adrenergic input to the heart and resembles the blunted effect of isoproterenol to increase HR in the TG^AC8^ heart *ex vivo* (Georget et al., 2002). Our results demonstrate that, although neither the abundance of transcripts coding for β-AR subtype proteins, nor Gαs, Gαi1, Gαi2, or Gαi3 proteins differ from those of WT, the abundance transcripts for genes that desensitize and internalize β-ARs (Koch et al., 2000) are increased (Supplementary Table S3). Protein levels of Arrb2, GRK5, and Dab_2_ also significantly exceed those in WT (Supplementary Table S1). The altered patterns of gene and protein expression in the TG^AC8^ SAN suggest that β-AR desensitization accounts, at least in part, for the reduced HR and contractility in response to β-AR stimulation. β-AR desensitization complements the reduction in catecholamines within TG^AC8^ mice, limiting the extent to which external adrenergic influences increase the high level of intracellular cAMP-PKA-Ca^2+^ signaling that result from overexpression of AC8 in the TG^AC8^ heart.

### Blunted Effectiveness of Vagal Input Into the TG^AC8^ SAN

Our results demonstrate that in addition to blunted adrenergic receptor signaling, parasympathetic inhibition has a minimal effect on the TG^AC8^. Although the reduction of basal high frequency power in TG^AC8^ might be interpreted to reflect reduced cholinergic receptor stimulation, it may be that muscarinic receptor signaling is exaggerated, but cannot overcome the extremely high AC8 induced cAMP-PKA-Ca^2+^ dependent signaling. Reductions in RGS signaling manifest as reduced transcription or overexpression of RGS2 and RGS6 in TG^AC8^ (Supplementary Tables S1, S3), are consistent with adaptations that promote receptor initiated Gi signaling, in an attempt to limit the markedly increased AC-cAMP signaling intrinsic to TG^AC8^ SAN cells.

In summary, our results provide evidence that the marked reduction to HRV in TG^AC8^ mice is minimally influenced by fluctuations in autonomic neuronal input to the SAN, and is instead driven by potent AC8 driven coupled clock mechanisms intrinsic to SAN cells. Because the overall pattern of marked coherency or loss of complexity within the TG^AC8^ heart rhythm is similar to that associated with aging or cardiac pathology (Iyengar et al., 1996; Fauchier et al., 1997; Huikuri and Stein, 2013; Yaniv et al., 2016), it will be crucial, in future studies, to determine whether TG^AC8^ will manifest a form of accelerated cardiac aging or heart failure.

### Significance and Opportunities for Further Scientific Advances

Our results show that in the context of high cAMP-PKA-Ca^2+^ signaling driven by mechanisms intrinsic to SAN cells, the TG^AC8^ mouse heart applies adaptive strategies in order to blunt additional external sympathetic input. These findings provide a segway to more detailed studies of these and other adaptations that partially disengage the heart from external adrenergic input with respect to prevention of arrhythmias, cell necrosis, apoptosis, and prevention of cardiomyopathy in response to chronically increased sympathetic stress. Such studies may provide novel bases for therapeutic intervention for arrythmias and heart failure.

The TG^AC8^ mouse, in which AC8 is overexpressed only in the heart, would appear to be a specifically valuable model to the emerging field of neurocardiology. One such adaptation utilized by TG^AC8^ to reduce sympathetic input to its heart is a reduction in circulating altered plasma catecholamines. In addition to altered hemodynamic signaling to the nervous system, afferent signals from heart, spinal cord to brain are thought to emerge via a network of autonomic ganglia embedded within heart epicardial tissue to alter catecholamines (Armour, 2008). Such retrograde neuronal signaling from heart to nervous system may be a crucial adaptation, in addition to altered hemodynamic or hormonal signals, generated from within the TG^AC8^ heart to repress production of brain catecholamines or reducing brain to heart, or brain to adrenal medulla signaling. This merits further study.

## Data Availability

All datasets generated for this study are included in the manuscript and/or the Supplementary Files.

## Ethics Statement

All studies were performed in accordance with the Guide for the Care and Use of Laboratory Animals published by the National Institutes of Health (NIH Publication no. 85-23, revised 1996). The experimental protocols were approved by the Animal Care and Use Committee of the National Institutes of Health (protocol #441-LCS-2016).

## Author Contributions

JM and MM performed all of the *in vivo* studies, aided in the collection and isolation of SAN, analyzed the data, and wrote the manuscript. CR aided in the collection analysis of *in vivo* data. KC performed the sample collection for RT-qPCR and catecholamine data analysis. YL performed the AC activity assays. YT performed all RT-qPCR tissue preparation and experiments and was responsible for all immunostaining of SAN tissue. KiT did the analysis. ST isolated and prepped SAN cells for staining and analysis. KT participated in analyses of mice ECGs *in vivo*. OM guided the initial *in vivo* HRV studies and design. CM designed the LME statistical method used for *in vivo* analysis. YY helped to develop the HRV analysis methods. TH and KP analyzed the blood catecholamine levels. IA designed the initial the study and oversaw the use and handling of animals involved. EL designed the research project, provided the materials and reagents, as well as aided in the writing of the manuscript.

## Conflict of Interest Statement

The authors declare that the research was conducted in the absence of any commercial or financial relationships that could be construed as a potential conflict of interest.

## References

[B1] Armour J. A. (2008). Potential clinical relevance of the ‘little brain’ on the mammalian heart. *Exp. Physiol.* 93 165–176. 10.1113/expphysiol.2007.041178 17981929

[B2] Behar J. A. Rosenberg A. A. Weiser-Bitoun I. Shemla O. Alexandrovich A. Konyukhov E. (2018). PhysioZoo: a novel open access platform for heart rate variability analysis of mammalian electrocardiographic data. *Front. Physiol.* 9:1390. 10.3389/fphys.2018.01390 30337883 PMC6180147

[B3] Ben-Ari M. Schick R. Barad L. Novak A. Ben-Ari E. Lorber A. (2014). From beat rate variability in induced pluripotent stem cell-derived pacemaker cells to heart rate variability in human subjects. *Heart Rhythm* 11 1808–1818. 10.1016/j.hrthm.2014.05.037 25052725 PMC4283811

[B4] Billman G. E. (2011). Heart rate variability - a historical perspective. *Front. Physiol.* 2:86. 10.3389/fphys.2011.00086 22144961 PMC3225923

[B5] Boyett M. Wang Y. D’Souza A. (2019). CrossTalk opposing view: heart rate variability as a measure of cardiac autonomic responsiveness is fundamentally flawed. *J. Physiol.* 597 2599–2601. 10.1113/JP277501 31006856 PMC6826226

[B6] Eisenhofer G. Goldstein D. S. Stull R. Keiser H. R. Sunderland T. Murphy D. L. (1986). Simultaneous liquid-chromatographic determination of 3,4-dihydroxyphenylglycol, catecholamines, and 3,4-dihydroxyphenylalanine in plasma, and their responses to inhibition of monoamine oxidase. *Clin. Chem.* 32 2030–2033. 3096593

[B7] Fauchier L. Babuty D. Cosnay P. Autret M. L. Fauchier J. P. (1997). Heart rate variability in idiopathic dilated cardiomyopathy: characteristics and prognostic value. *J. Am. Coll. Cardiol.* 30 1009–1014. 10.1016/s0735-1097(97)00265-9 9316532

[B8] Georget M. Mateo P. Vandecasteele G. Jurevicius J. Lipskaia L. Defer N. (2002). Augmentation of cardiac contractility with no change in L-type Ca2+ current in transgenic mice with a cardiac-directed expression of the human adenylyl cyclase type 8 (AC8). *FASEB J.* 16 1636–1638. 10.1096/fj.02-0292fje 12206999

[B9] Goldberger A. L. (1991). Is the normal heartbeat chaotic or homeostatic? *News Physiol. Sci.* 6 87–91. 10.1152/physiologyonline.1991.6.2.87 11537649

[B10] Goldberger A. L. Amaral L. A. Hausdorff J. M. Ivanov P. Peng C. K. Stanley H. E. (2002). Fractal dynamics in physiology: alterations with disease and aging. *Proc. Natl. Acad. Sci. U.S.A.* 99 (Suppl. 1) 2466–2472. 10.1073/pnas.012579499 11875196 PMC128562

[B11] Huang M. H. Friend D. S. Sunday M. E. Singh K. Haley K. Austen K. F. (1996). An intrinsic adrenergic system in mammalian heart. *J. Clin. Invest.* 98 1298–1303. 10.1172/JCI118916 8823294 PMC507555

[B12] Huikuri H. V. Stein P. K. (2013). Heart rate variability in risk stratification of cardiac patients. *Prog. Cardiovasc. Dis.* 56 153–159. 10.1016/j.pcad.2013.07.003 24215747

[B13] Iyengar N. Peng C. K. Morin R. Goldberger A. L. Lipsitz L. A. (1996). Age-related alterations in the fractal scaling of cardiac interbeat interval dynamics. *Am. J. Physiol.* 271(4 Pt 2), R1078–R1084. 10.1152/ajpregu.1996.271.4.R1078 8898003

[B14] Koch W. J. Lefkowitz R. J. Rockman H. A. (2000). Functional consequences of altering myocardial adrenergic receptor signaling. *Annu. Rev. Physiol.* 62 237–260. 10.1146/annurev.physiol.62.1.237 10845091

[B15] Kuznetsova A. Brockhoff P. B. Christensen R. H. B. (2016). *lmerTest: Tests in Linear Mixed Effects Models. R package version* 2.0-33. Available at: https://CRAN.R-project.org/package=lmerTest (accessed February 11, 2019).

[B16] Lahiri M. K. Kannankeril P. J. Goldberger J. J. (2008). Assessment of autonomic function in cardiovascular disease: physiological basis and prognostic implications. *J. Am. Coll. Cardiol.* 51 1725–1733. 10.1016/j.jacc.2008.01.038 18452777

[B17] Lakatta E. G. Maltsev V. A. Vinogradova T. M. (2010). A coupled SYSTEM of intracellular Ca2+ clocks and surface membrane voltage clocks controls the timekeeping mechanism of the heart’s pacemaker. *Circ. Res.* 106 659–673. 10.1161/CIRCRESAHA.109.206078 20203315 PMC2837285

[B18] Lipskaia L. Defer N. Esposito G. Hajar I. Garel M. C. Rockman H. A. (2000). Enhanced cardiac function in transgenic mice expressing a Ca(2+)-stimulated adenylyl cyclase. *Circ. Res.* 86 795–801. 10.1161/01.res.86.7.795 10764414

[B19] Malik M. Hnatkova K. Huikuri H. V. Lombardi F. Schmidt G. Zabel M. (2019). Cross talk proposal: heart rate variability is a valid measure of cardiac autonomic responsiveness. *J. Physiol.* 597 2595–2598. 10.1113/JP277500 31006862 PMC6826215

[B20] Mandel Y. Weissman A. Schick R. Barad L. Novak A. Meiry G. (2012). Human embryonic and induced pluripotent stem cell-derived cardiomyocytes exhibit beat rate variability and power-law behavior. *Circulation* 125 883–893. 10.1161/CIRCULATIONAHA.111.045146 22261196 PMC3697086

[B21] Mangoni M. E. Nargeot J. (2008). Genesis and regulation of the heart automaticity. *Physiol. Rev.* 88 919–982. 10.1152/physrev.00018.2007 18626064

[B22] McCraty R. Zayas M. A. (2014). Cardiac coherence, self-regulation, autonomic stability, and psychosocial well-being. *Front. Psychol.* 5:1090. 10.3389/fpsyg.2014.01090 25324802 PMC4179616

[B23] Missale C. Nash S. R. Robinson S. W. Jaber M. Caron M. G. (1998). Dopamine receptors: from structure to function. *Physiol. Rev.* 78 189–225. 10.1152/physrev.1998.78.1.189 9457173

[B24] Monfredi O. Lyashkov A. E. Johnsen A. B. Inada S. Schneider H. Wang R. (2014). Biophysical characterization of the underappreciated and important relationship between heart rate variability and heart rate. *Hypertension* 64 1334–1343. 10.1161/HYPERTENSIONAHA.114.03782 25225208 PMC4326239

[B25] Monfredi O. Maltseva L. A. Spurgeon H. A. Boyett M. R. Lakatta E. G. Maltsev V. A. (2013). Beat-to-Beat variation in periodicity of local calcium releases contributes to intrinsic variations of spontaneous cycle length in isolated single sinoatrial node cells. *PLoS One* 8:e67247. 10.1371/journal.pone.0067247 23826247 PMC3695077

[B26] Peoples J. Maxmillan T. Le Q. Nadochiy S. Brookes P. Porter G. (2018). Metabolomics reveals critical adrenergic regulatory checkpoints in glycolysis and pentose-phosphate pathways in embryonic heart. *J. Biol. Chem.* 293 6925–6941. 10.1074/jbc.RA118.002566 29540484 PMC5936816

[B27] R Core Development Team (2017). *R: A Language and Environment for Statistical Computing.* Vienna: R Foundation for Statistical Computing.

[B28] Rocchetti M. Malfatto G. Lombardi F. Zaza A. (2000). Role of the input/output relation of sinoatrrial myocytes in cholinergic modulation of heart rate variability. *J. Cardiovasc. Electrophysiol.* 11 522–530. 10.1111/j.1540-8167.2000.tb00005.x 10826931

[B29] RStudio Team (2017). *RStudio: Integrated Development for R.* Boston, MA: RStudio, Inc.

[B30] Smith R. Thayer J. F. Khalsa S. S. Lane R. D. (2017). The hierarchical basis of neurovisceral integration. *Neurosci. Biobehav. Rev.* 75 274–296. 10.1016/j.neubiorev.2017.02.003 28188890

[B31] Thayer J. F. Yamamoto S. S. Brosschot J. F. (2010). The relationship of autonomic imbalance, heart rate variability and cardiovascular disease risk factors. *Int. J. Cardiol.* 141 122–131. 10.1016/j.ijcard.2009.09.543 19910061

[B32] Thireau J. Zhang B. L. Poisson D. Babuty D. (2008). Heart rate variability in mice: a theoretical and practical guide. *Exp. Physiol.* 93 83–94. 10.1113/expphysiol.2007.040733 17911354

[B33] Thomas S. A. Matsumoto A. M. Palmiter R. D. (1995). Noradrenaline is essential for mouse fetal development. *Nature* 374 643–646. 10.1038/374643a0 7715704

[B34] Tsutsui K. Monfredi O. J. Sirenko-Tagirova S. G. Maltseva L. A. Bychkov R. Kim M. S. (2018). A coupled-clock system drives the automaticity of human sinoatrial nodal pacemaker cells. *Sci. Signal* 11:eaa7608 10.1126/scisignal.aap7608 PMC6138244 29895616

[B35] Vinogradova T. M. Lyashkov A. E. Zhu W. Ruknudin A. M. Sirenko S. Yang D. (2006). High basal protein kinase A-dependent phosphorylation drives rhythmic internal Ca2+ store oscillations and spontaneous beating of cardiac pacemaker cells. *Circ. Res.* 98 505–514. 10.1161/01.RES.0000204575.94040.d1 16424365

[B36] Vinogradova T. M. Sirenko S. Lyashkov A. E. Younes A. Li Y. Zhu W. (2008). Constitutive phosphodiesterase activity restricts spontaneous beating rate of cardiac pacemaker cells by suppressing local Ca2+ releases. *Circ. Res.* 102 761–769. 10.1161/CIRCRESAHA.107.161679 18276917

[B37] Wang Y. Lin W. K. Crawford W. Ni H. Bolton E. L. Khan H. (2017). Optogenetic control of heart rhythm by selective stimulation of cardiomyocytes derived from Pnmt+ cells in murine heart. *Sci. Rep.* 7:40687. 10.1038/srep40687 28084430 PMC5234027

[B38] Yang D. Lyashkov A. E. Li Y. Ziman B. D. Lakatta E. G. (2012). RGS2 overexpression or G(i) inhibition rescues the impaired PKA signaling and slow AP firing of cultured adult rabbit pacemaker cells. *J. Mol. Cell. Cardiol.* 53 687–694. 10.1016/j.yjmcc.2012.08.007 22921807 PMC3472119

[B39] Yang J. Huang J. Maity B. Gao Z. Lorca R. A. Gudmundsson H. (2010). RGS6, a modulator of parasympathetic activation in heart. *Circ. Res.* 107 1345–1349. 10.1161/CIRCRESAHA.110.224220 20864673 PMC2997524

[B40] Yaniv Y. Ahmet I. Liu J. Lyashkov A. E. Guiriba T. R. Okamoto Y. (2014a). Synchronization of sinoatrial node pacemaker cell clocks and its autonomic modulation impart complexity to heart beating intervals. *Heart Rhythm* 11 1210–1219. 10.1016/j.hrthm.2014.03.049 24713624 PMC4065846

[B41] Yaniv Y. Lyashkov A. E. Sirenko S. Okamoto Y. Guiriba T. R. Ziman B. D. (2014b). Stochasticity intrinsic to coupled-clock mechanisms underlies beat-to-beat variability of spontaneous action potential firing in sinoatrial node pacemaker cells. *J. Mol. Cell. Cardiol.* 77 1–10. 10.1016/j.yjmcc.2014.09.008 25257916 PMC4312254

[B42] Yaniv Y. Ahmet I. Tsutsui K. Behar J. Moen J. M. Okamoto Y. (2016). Deterioration of autonomic neuronal receptor signaling and mechanisms intrinsic to heart pacemaker cells contribute to age-associated alterations in heart rate variability in vivo. *Aging Cell* 15 716–724. 10.1111/acel.12483 27168363 PMC4933656

[B43] Yaniv Y. Sirenko S. Ziman B. D. Spurgeon H. A. Maltsev V. A. Lakatta E. G. (2013). New evidence for coupled clock regulation of the normal automaticity of sinoatrial nodal pacemaker cells: bradycardic effects of ivabradine are linked to suppression of intracellular Ca(2)(+) cycling. *J. Mol. Cell. Cardiol.* 62 80–89. 10.1016/j.yjmcc.2013.04.026 23651631 PMC3735682

[B44] Younes A. Lyashkov A. E. Graham D. Sheydina A. Volkova M. V. Mitsak M. (2008). Ca(2+) -stimulated basal adenylyl cyclase activity localization in membrane lipid microdomains of cardiac sinoatrial nodal pacemaker cells. *J. Biol. Chem.* 283 14461–14468. 10.1074/jbc.M707540200 18356168 PMC2386925

[B45] Zahanich I. Sirenko S. G. Maltseva L. A. Tarasova Y. S. Spurgeon H. A. Boheler K. R. (2011). Rhythmic beating of stem cell-derived cardiac cells requires dynamic coupling of electrophysiology and Ca cycling. *J. Mol. Cell. Cardiol.* 50 66–76. 10.1016/j.yjmcc.2010.09.018 20920509 PMC3018669

[B46] Zaza A. Lombardi F. (2001). Autonomic indexes based on the analysis of heart rate variability: a view from the sinus node. *Cardiovasc. Res.* 50 434–442. 10.1016/s0008-6363(01)00240-1 11376619

