# Supplementary material for: Overexpression of a Neuronal Type Adenylyl Cyclase (Type 8) in Sinoatrial Node Markedly Impacts Heart Rate and Rhythm
Source: Front Neurosci. 2019 Jun 18;13:615. doi: 10.3389/fnins.2019.00615 (PMC6591434; doi:10.3389/fnins.2019.00615)
Supplement: Supplementary file 1 [file Data_Sheet_1.PDF]

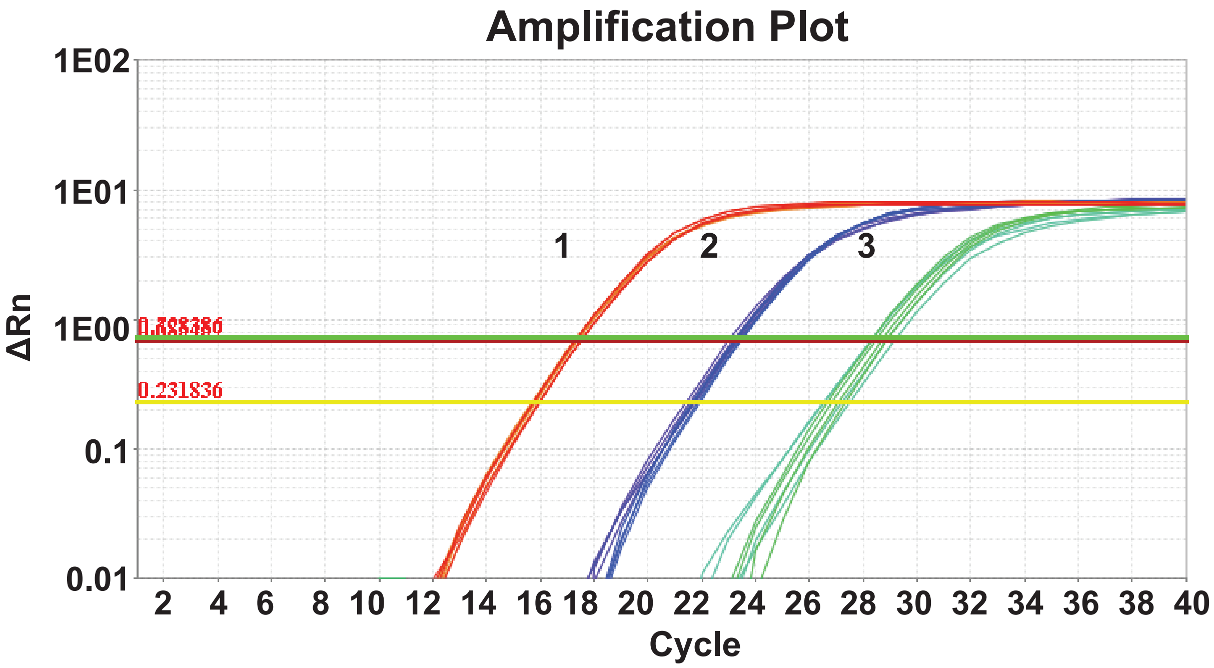

Supplemental Figure 1. Expression analysis of TG and endogenous ADCY8 normalized to HPRT in transgene and wild type mice. Amplification plot of RT-qPCR. 1-Hs. ADCY8 (human) expressed only in TG (N=3); 2-HPRT (housekeeping gene) expressed in WT(N=3) and TG (N=3); 3-Mm.ADCY8 (endogenous) expressed in WT (N=3) and TG (N=3).

Supplemental figure 1

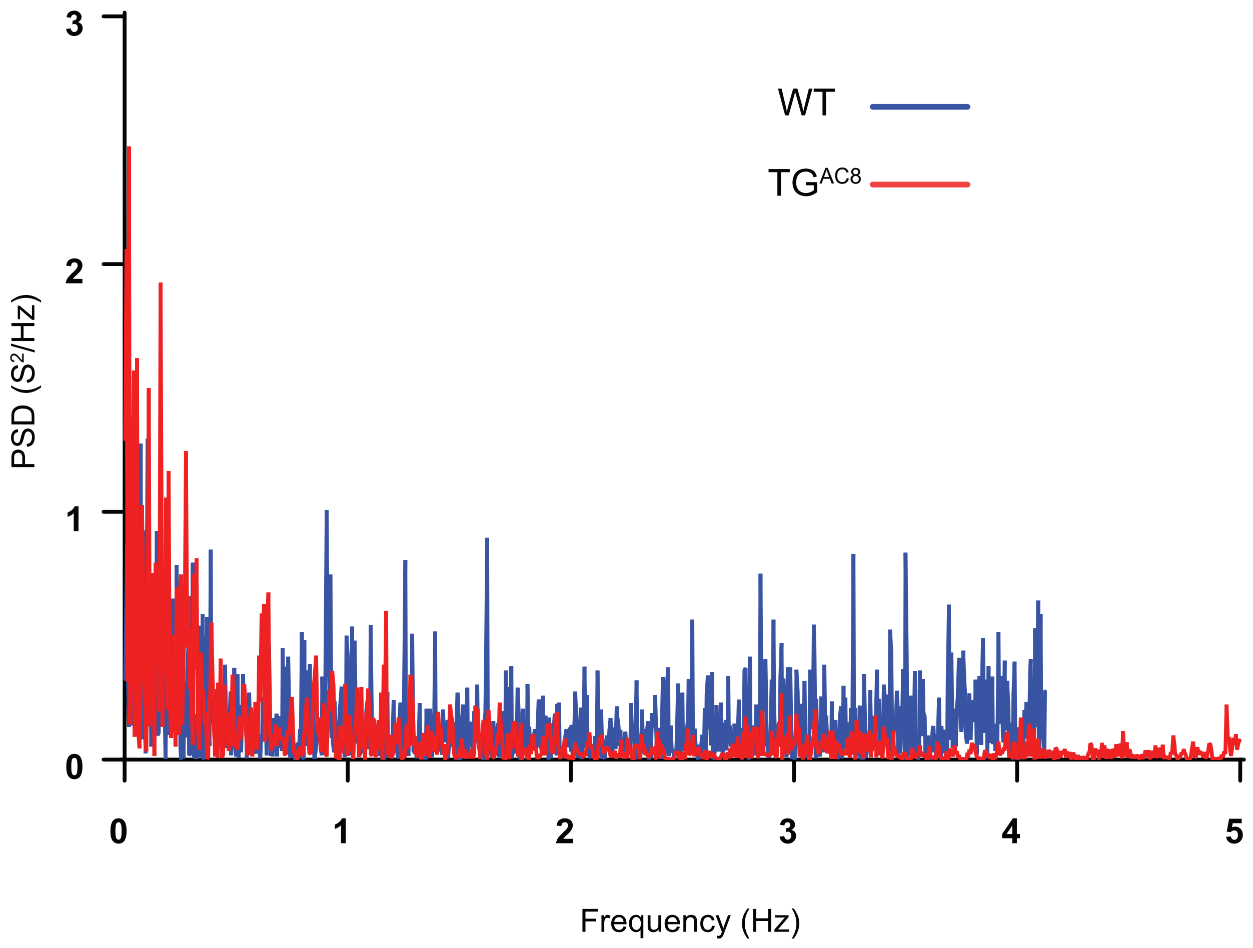

Supplemental Figure 2. Representative power spectrum plots for WT and TG^{AC8} mice.

Supplemental figure 2

Supplemental Table 1. Quantification of immunofluorescence

Antibody	TG^{AC8}			WT			P-value	TG^{AC8}/WT
	N	CTCF	STE	N	CTCF	STE		
hADCY8	7	8,795,437	2,587,771	9	1,526,010	407,447	0.012	5.764
GRK5	8	26,374,821	4,486,725	9	13,255,600	1,400,266	0.041	1.990
Arrb2	8	1.0600	0.171	8	0.685	0.089	0.078	1.536
DAB2	8	10,950,414	1,972,448	7	3,935,052	1,689,600	0.046	2.783
RGS6	9	1,364,939	120,331	7	13,032,476	5,115,687	0.009	0.105
TH	7	35,941,055	7,976,877	8	10,607,057	1,906,936	0.019	3.388
DBH	17	1.130	0.232	16	3.197	1.434	0.017	0.354
HCN4	30	1,260,319	29,771,697	30	1,332,994.6	223,775.2	0.366	0.945

Supplemental Table 1. Quantification of immunolabeling of TG^{AC8} and WT SANC. Ratio of relative abundance of TG^{AC8} to WT is calculated in the final column.

Supplemental Table 2. HRV analyses

Variable		Dual Autonomic Block				
		WT	TG	F-Value	P-value	Pairwise Interactions
Time Domain	SDRR	2.00 ± 0.63Θ	0.66 ± 0.24	20.546	0.001	WT Basal X WT Dual Autonomic Block, WT Basal X TG Basal
	CV	1.59 ± 0.48Θ	0.72 ± 0.24	14.007	0.004	WT Basal X WT Dual Autonomic Block, WT Basal X TG Basal
	Mean RR ‡	124.8 ± 9.13	91.54 ± 6.9	3.337	0.098	
	Min RR	119.4 ± 8.42*Θ	89.74 ± 6.75Θ	5.561	0.040	WT Basal X WT Dual Autonomic Block, TG Basal X TG Dual Autonomic Block, WT Basal X TG Basal, WT Dual Autonomic Block X TG Dual Autonomic Block
	Max RR	129.8 ± 10.10*Θ	93.18 ± 7.30	19.571	0.001	WT Basal X WT Dual Autonomic Block, WT Basal X TG Basal, WT Dual Autonomic Block X TG Dual Autonomic Block
	Range RR	10.38 ± 3.91Θ	3.44 ± 1.17	30.224	0.000	WT Basal X WT Dual Autonomic Block, WT Basal X TG Basal
Frequency Domain	VLF (ms^2/Hz) ‡	142.71 ± 75.9	48.72 ± 33.6	0.545	0.477	
	LF (ms2/Hz)	14.44 ± 5.79Θ	6.74 ± 4.76	14.416	0.004	WT Basal X WT Dual Autonomic Block, WT Basal X TG Basal
	HF (ms2/Hz)	53.71 ± 22.63Θ	20.58 ± 11.38	5.184	0.046	WT Basal X WT Dual Autonomic Block, WT Basal X TG Basal
	Total Power (ms2/Hz) ‡	210.87 ± 64.26	76.04 ± 31.33	2.599	0.138	
Non-linear	SD1	1.34 ± 0.67Θ	0.40 ± 0.28	24.921	0.001	WT Basal X WT Dual Autonomic Block, WT Basal X TG Basal
	SD2	2.43 ± 0.82Θ	0.82 ± 0.31	17.884	0.002	WT Basal X WT Dual Autonomic Block, WT Basal X TG Basal
	SD1:2 ‡	0.58 ± 0.26	0.53 ± 0.30	0.293	0.600	
	MSE (E1)	0.42 ± 0.12*Θ	0.08 ± 0.12Θ	119.116	0.000	WT Basal X WT Dual Autonomic Block, TG Basal X TG Dual Autonomic Block, WT Basal X TG Basal, WT Dual Autonomic Block X TG Dual Autonomic Block

Variable		Parasympathetic Block				
		WT	TG	F-Value	P-value	Pairwise Interactions
Time Domain	SDRR ‡ Ŧ	1.82 ± 0.83	0.68 ± 0.25	3.336	0.101	
	CV Ŧ	1.82 ± 0.77	0.81 ± 0.27	1.028	0.337	
	Mean RR	99.38 ± 4.65*Θ	84.54 ± 2.70Θ	10.884	0.009	WT Basal X WT Parasympathetic Block, TG Basal X TG Parasympathetic Block, WT Basal X TG Basal, WT Parsympathetic Block X TG Parsympathetic Block
	Min RR	94.83 ± 3.57*Θ	82.96 ± 2.34	5.569	0.043	WT Basal X WT Parasympathetic Block, WT Basal X TG Basal, WT Parsympathetic Block X TG Parsympathetic Block
	Max RR	103.68 ± 5.94*Θ	86.09 ± 3.08Θ	8.970	0.015	WT Basal X WT Parasympathetic Block, TG Basal X TG Parasympathetic Block, WT Basal X TG Basal, WT Parsympathetic Block X TG Parsympathetic Block
	Range RR ‡ Ŧ	8.84 ± 3.93	3.13 ± 1.04	4.129	0.073	
Frequency Domain	VLF (ms^2/Hz) ‡	99.77 ± 41.7*	15.53 ± 13.0	0.275	0.612	
	LF (ms2/Hz)	5.466 ± 1.85*Θ	3.79 ± 1.80	5.627	0.042	WT Basal X WT Parasympathetic Block, WT Basal X TG Basal
	HF (ms2/Hz) ‡ Ŧ	13.67 ± 6.44	17.26 ± 8.86	4.399	0.065	
	Total Power (ms2/Hz) ‡ Ŧ	118.91 ± 47.30	36.59 ± 15.48	2.338	0.161	
Non-linear	SD1	0.32 ± 0.11Θ	0.56 ± 0.25	7.367	0.024	WT Basal X WT Parasympathetic Block, WT Basal X TG Basal
	SD2 ‡ Ŧ	2.56 ± 1.17	0.78 ± 0.28Θ	2.035	0.187	
	SD1:2 Ŧ	0.13 ± 0.04	0.69 ± 0.24	24.527	0.001	
	MSE (E1)	0.10 ± 0.06Θ	0.15 ± 0.12Θ	26.181	0.001	WT Basal X WT Parasympathetic Block, TG Basal X TG Parasympathetic Block, WT Basal X TG Basal

Variable		Sympathetic Block				
		WT	TG	F-Value	P-value	Pairwise Interactions
Time Domain	SDRR ‡ Ŧ	5.77 ± 3.20*	2.02 ± 0.25	3.820	0.066	
	CV ‡ Ŧ	3.97 ± 2.04*	2.08 ± 0.27	2.971	0.102	
	Mean RR ‡	140.8 ± 14.6*	94.69 ± 2.70	0.051	0.826	
	Min RR	126.56 ± 9.40*Θ	89.37 ± 2.34Θ	25.866	0.001	WT Basal X WT Sympathetic Block, TG Basal X TG Sympathetic Block, WT Basal X TG Basal, WT Sympathetic Block X TG Sympathetic Block
	Max RR ‡	160.60 ± 31.7*	100.53 ± 3.08	0.029	0.868	
	Range RR ‡ Ŧ	34.04 ± 25.92*	11.15 ± 1.04	0.955	0.341	
Frequency Domain	VLF (ms^2/Hz) ‡	96.66 ± 71.3*	22.21 ± 13.0	0.243	0.634	
	LF (ms2/Hz) ‡	50.49 ± 27.2*	10.58 ± 1.80	0.042	0.839	
	HF (ms2/Hz) ‡	88.19 ± 54.00*	21.19 ± 8.86	0.533	0.475	
	Total Power (ms2/Hz) ‡	235.35 ± 144.5*	54.00 ± 15.48	0.220	0.645	
Non-linear	SD1 ‡ Ŧ	4.53 ± 3.24*	1.19 ± 0.25	4.186	0.056	
	SD2 ‡ Ŧ	6.66 ± 3.46*	2.58 ± 0.28	3.647	0.072	
	SD1:2	0.63 ± 0.28	0.51 ± 0.24	0.647	0.442	
	MSE (E1) ‡ Ŧ	1.27 ± 0.69*	0.50 ± 0.12	3.670	0.071	

Variable		β-Adrenergic Stimulation				
		WT	TG	F-Value	P-value	Pairwise Interactions
Time Domain	SDRR ‡ Ŧ	0.77 ± 0.11	0.59 ± 0.12	3.352	0.082	
	CV ‡ Ŧ	0.94 ± 0.16	0.75 ± 0.16	2.194	0.154	
	Mean RR	82.90 ± 4.42Θ	78.48 ± 1.47Θ	14.157	0.004	WT Basal X WT β-Adrenergic, TG Basal X TG β-Adrenergic
	Min RR	80.48 ± 5.01Θ	76.71 ± 1.70	15.433	0.001	WT Basal X WT β-Adrenergic, WT Basal X TG Basal
	Max RR	85.61 ± 4.08Θ	80.47 ± 1.25Θ	10.683	0.008	WT Basal X WT β-Adrenergic, TG Basal X TG β-Adrenergic, WT Basal X TG Basal
	Range RR ‡ Ŧ	5.12 ± 2.61	3.76 ± 0.82	2.694	0.116	
Frequency Domain	VLF (ms^2/Hz)	6.238 ± 7.73*	4.271 ± 2.31Θ	8.889	0.007	WT Basal X WT β-Adrenergic, WT Basal X TG Basal
	LF (ms2/Hz) Ŧ	3.94 ± 3.61	5.34 ± 3.88	1.575	0.224	
	HF (ms2/Hz) ‡ Ŧ	8.317 ± 8.93	8.059 ± 6.77	3.723	0.068	
	Total Power (ms2/Hz)	18.50 ± 20.08Θ	17.67 ± 12.72Θ	9.019	0.007	WT Basal X WT β-Adrenergic, TG Basal X TG β-Adrenergic
Non-linear	SD1 ‡ Ŧ	0.63 ± 0.17	0.52 ± 0.16	2.733	0.114	
	SD2 ‡ Ŧ	0.89 ± 0.15	0.64 ± 0.10	3.169	0.090	
	SD1:2	0.72 ± 0.22	0.81 ± 0.22	0.718	0.407	
	MSE (E1) ‡ Ŧ	0.15 ± 0.06	0.08 ± 0.06	4.304	0.051	

Variable		Basal	
		WT	TG
Time Domain	SDRR	10.89 ± 4.17*	3.40 ± 4.28
	CV	8.05 ± 3.03*	3.25 ± 3.46
	Mean RR	134.85 ± 9.45*	93.49 ± 14.1
	Min RR	110.4 ± 8.70*	84.88 ± 8.71
	Max RR	159.7 ± 13.99*	103.15 ± 22.0
	Range RR	49.35 ± 15.12*	18.27 ± 19.11
Frequency Domain	VLF (ms^2/Hz)	111.90 ± 67.4*	26.52 ± 31.5
	LF (ms2/Hz)	54.51 ± 19.7*	17.96 ± 22.2
	HF (ms2/Hz)	81.67 ± 37.01*	31.58 ± 24.64
	Total Power (ms2/Hz)	248.09 ± 67.02*	76.07 ± 70.78
Non-linear	SD1	8.53 ± 4.19*	2.22 ± 3.19
	SD2	12.65 ± 4.64*	4.21 ± 5.19
	SD1:2	0.66 ± 0.21*	0.50 ± 0.17
	MSE (E1)	1.98 ± 0.37*	0.64 ± 0.62

Supplemental Table 2. Top, listing of mean and standard error of the measured HRV parameters. ‡ $p<0.05$ for main effects genotype differences. Ŧ $p<0.05$ for main effects of drug differences. If a significant interaction effect was found, as determined by a linear mixed effects model with difference of least squares means post hoc test and Satterthwaite's approximation for degrees of freedom, then * $p<0.05$ for significant pairwise genotype differences, and Θ $p<0.05$ for drug differences. F-value and p-value for interactions has been provided for all samples in the table below.

Supplemental Table 3. Analysis of select transcripts within SAN

Gene	RQ TG^{AC8}	STE TG^{AC8}	RQ WT	STE WT	P-value	TG^{AC8}/WT
ADRB1	0.931	0.075	0.984	0.041	0.582	0.946
ADRB2	1.213	0.049	1.196	0.095	0.882	1.015
ADRB3	0.855	0.121	0.752	0.078	0.528	1.137
Arrb2	1.325	0.061	1.047	0.052	0.005	1.266
Dab2	1.326	0.073	0.946	0.048	0.003	1.401
GNAI2	0.985	0.003	0.998	0.009	0.259	0.987
GNAI3	1.012	0.008	1.020	0.030	0.846	0.992
GNG2	0.870	0.038	0.996	0.041	0.046	0.874
GNG5	1.025	0.047	1.003	0.043	0.110	1.022
GNAS	1.691	0.318	1.092	0.185	0.168	1.549
GRK2	1.029	0.043	0.983	0.037	0.473	1.047
GRK5	1.955	0.181	1.105	0.090	0.003	1.769
PNMT	0.710	0.079	1.090	0.082	0.012	0.651
RGS2	0.755	0.031	1.166	0.069	0.001	0.648
RGS6	0.866	0.095	1.004	0.042	0.295	0.863

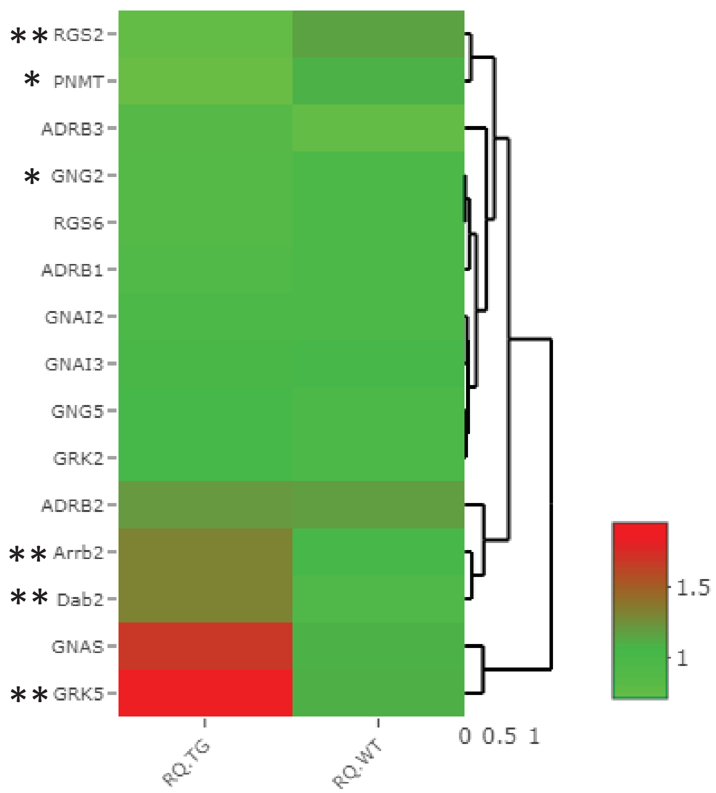

Supplemental Table 3. Abundance of transcripts for selected genes in TG^{AC8} and WT mice determined by RT-qPCR. Mean and STE: and p-value by Student's t-test are presented. The last column shows the ratio of TG^{AC8} to WT. Heat map represents relative quantification (RQ) of each assessed transcript. *p<0.05, ** p<0.01. Note significant upregulation of Arrb2, Dab2, GRK5 and downregulation of RGS2, PNMT, and GNG2 in TG^{AC8} samples.

Supplemental Table 4. List of primers used in this study

Primer Name	Sequence	Amplicon Size
Hs.ADCY8-FW	CTTCGATGAGTTGCTTGGTGAAG	110
Hs.ADCY8-RV	ACATTGCTGTTTTTCAGGTGACA	
Mm.ADCY8-FW	GAAGTGAAGGGAATGGGAATTGT	114
Mm.ADCY8-RV	GAAGATCGTGGCGCTGCTA	
Adrb1-FW	GCCCACAGATCTATCGAATCATC	90
Adrb1-RV	CTCCCAACTCCTCCTAAACTTTCC	
Adrb2-FW	AAGAGCACAAAGCCCTCAAGAC	90
Adrb2-RV	CGTGCACGATATTGACAATGAAG	
Adrb3-FW	CTGCGCACCTTAGGTCTCATTAT	116
Adrb3-RV	ATGAAAACTCCGCTGGGAACTAG	
Arrb2-FW	GGAGCCCCTTAATGTCAACGT	91
Arrb2-RV	AATGTCGGCGTACTGTCTCACA	
Dab2-FW	GGGATTGGCTGGTATCAGTGA	81
Dab2-RV	GCCATTGGTTGTGCTTGTTTCT	
Gnai2-RV	CCAGCTGCTTCTCCCATCTT	80
Gnai2-FW	CAACCCCTCCCCCAAGTCTA	
Gnai3-FW	TAAACAGCATTCACCTTGGAGAGT	93
Gnai3-RV	GGTTAACCCCGACAAATACAACTT	
Gng2-FW	TGGCTGTCCTGGAACTTGTTC	92
Gng2-RV	TGGTGGCTCATGCCTTTATTC	
Gng5-FW	GCAGCTGCAGACTTGAAACAGT	88
Gng5-RV	TGAAGGGATTCGTACTTGAAGACA	
Gnas-FW	AACATCCGCCGTGTCTTCA	83
Gnas-RV	TTCCCTTCTTAGAGCAGCTCGTATT	
Grk2-FW	GCCTATGACAGCAGTGCAGACT	113
Grk2-RV	CGGTCAATCTCATGCTTGTCTTT	
Grk5-FW	AGAAAGGGCTGTTCCACAGACT	88
Grk5-RV	CGGTGGTTACAACTGGTCTTAGG	
Pnmt-FW	CTCCGGCCCCACCATATATC	91
Pnmt-RV	CTCCTGACGGTTGACTTCCAA	
Rgs2-FW	AATCTCCTTTGCCATTAAGTTCCA	89
Rgs2-RV	CAGGCATGGTGGCATCTTCTAT	
Rgs6-FW	AGTACCCAGGAAGGGTCTCTTACA	111
Rgs6-RV	ATCCTGTTGGTGAGGTAGTTTGAAC	
Hprt-FW	CTTCCTCCTCAGACCGCTTTT	97
Hprt-RV	CATAACCTGGTTCATCATCGCTAA	

Supplemental Table 4. Primers and amplicon size for RT-qPCR analyses performed on RNA from SAN tissue.
